# Differences in coagulopathy indices in patients with severe versus non-severe COVID-19: a meta-analysis of 35 studies and 6427 patients

**DOI:** 10.1038/s41598-021-89967-x

**Published:** 2021-05-17

**Authors:** Alberto Polimeni, Isabella Leo, Carmen Spaccarotella, Annalisa Mongiardo, Sabato Sorrentino, Jolanda Sabatino, Salvatore De Rosa, Ciro Indolfi

**Affiliations:** 1 Division of Cardiology, Department of Medical and Surgical Sciences, Magna Graecia University, Catanzaro, Italy; 2 Department of Medical and Surgical Sciences, Center for Cardiovascular Research, Magna Graecia University, Catanzaro, Italy; 3 Mediterranea Cardiocentro, Naples, Italy

**Keywords:** Cardiology, Infectious diseases

## Abstract

Coronavirus disease 2019 (COVID-19) is a highly contagious disease that appeared in China in December 2019 and spread rapidly around the world. Several patients with severe COVID-19 infection can develop a coagulopathy according to the ISTH criteria for disseminated intravascular coagulopathy (DIC) with fulminant activation of coagulation, resulting in widespread microvascular thrombosis and consumption of coagulation factors. We conducted a meta-analysis in order to explore differences in coagulopathy indices in patients with severe and non-severe COVID-19. An electronic search was performed within PubMed, Google Scholar and Scopus electronic databases between December 2019 (first confirmed Covid-19 case) up to April 6th, 2020. The primary endpoint was the difference of D-dimer values between Non-Severe vs Severe disease and Survivors vs Non-Survivors. Furthermore, results on additional coagulation parameters (platelet count, prothrombin time, activated partial thromboplastin time) were also analyzed. The primary analysis showed that mean d-dimer was significantly lower in COVID-19 patients with non-severe disease than in those with severe (SMD − 2.15 [− 2.73 to − 1.56], I^2^ 98%, P < 0.0001). Similarly, we found a lower mean d-dimer in Survivors compared to Non-Survivors (SMD − 2.91 [− 3.87 to − 1.96], I^2^ 98%, P < 0.0001). Additional analysis of platelet count showed higher levels of mean PLT in Non-Severe patients than those observed in the Severe group (SMD 0.77 [0.32 to 1.22], I^2^ 96%, P < 0.001). Of note, a similar result was observed even when Survivors were compared to Non-Survivors (SMD 1.84 [1.16 to 2.53], I^2^ 97%, P < 0.0001). Interestingly, shorter mean PT was found in both Non-Severe (SMD − 1.34 [− 2.06 to − 0.62], I^2^ 98%, P < 0.0002) and Survivors groups (SMD − 1.61 [− 2.69 to − 0.54], I^2^ 98%, P < 0.003) compared to Severe and Non-Survivor patients. In conclusion, the results of the present meta-analysis demonstrate that Severe COVID-19 infection is associated with higher D-dimer values, lower platelet count and prolonged PT. This data suggests a possible role of disseminated intravascular coagulation in the pathogenesis of COVID-19 disease complications.

## Introduction

Coronavirus Disease 2019 (COVID-19), caused by a novel coronavirus (SARS-CoV-2), is a highly contagious disease that appeared in Wuhan, Hubei province of China in December 2019 and spread rapidly in China and even around the world^1^.

Most of the infected patients have mild symptoms including fever, fatigue and cough. Nevertheless, in severe cases, patients can progress rapidly and develop the acute respiratory distress syndrome, septic shock, metabolic acidosis and coagulopathy^2^.

**Figure 1:**
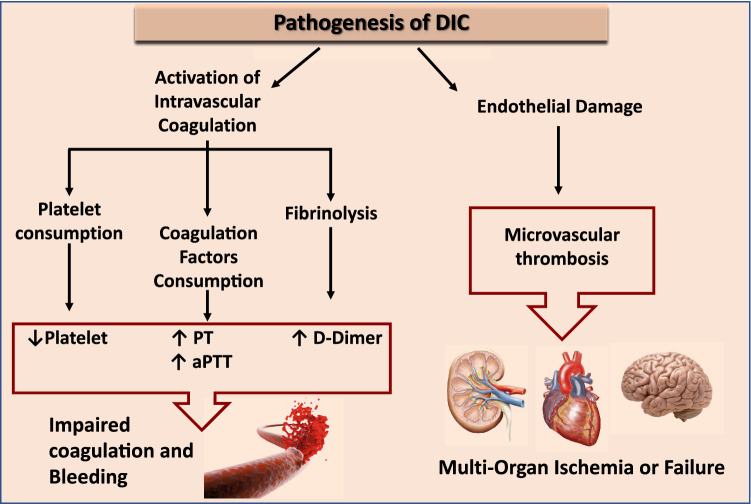
Pathogenesis of disseminated intravascular coagulation. DIC is characterized by systemic activation of blood coagulation, which results in generation and deposition of fibrin, leading to microvascular thrombi contributing to multi-organ dysfunction. Furthermore, consumption of clotting factors and platelets can result in life-threatening hemorrhage.

Although COVID-19 has a relatively low mortality rate, it can be highly deadly and lethal, especially in high-risk patients, and to date, there is no specific treatment available for this new disease. Therefore, it is mandatory to identify potential risk factors for predicting disease progression and severity. Coagulation abnormalities have been already detected in other severe coronavirus infections. Prolonged activated partial-thromboplastin time, elevated D-dimer and thrombocytopenia have been described in patients with SARS-CoV1. Moreover, even if less data is available about MERS-CoV, DIC was often associated with fatal cases of this very severe form of pneumonia^3^. Similarly, several patients with severe COVID-19 infection can develop a coagulopathy according to the International Society on Thrombosis and Haemostasis (ISTH) criteria for disseminated intravascular coagulopathy (DIC) with fulminant activation of coagulation^4^, resulting in widespread microvascular thrombosis and consumption of coagulation factors (Fig. 1).

SARS-CoV-2 infection has been in fact associated with pulmonary embolism^5^, deep vein thrombosis and microthrombi formation^6^. The pro-inflammatory environment resulting from the infection causes an endothelial dysfunction that could be responsible of an imbalance between pro-thrombotic and anti-thrombotic factors. The resulting hyper thrombotic state carries unique hallmarks, in a certain grade overlapping with DIC, that need to be fully discovered yet. The attention on this aspect is so high that it has been postulated that the COVID-19, previously considered mainly as a respiratory disease, could be instead defined in his most severe form an endothelial disease^7^.

D-dimer is a soluble fibrin degradation product deriving from the plasmin-mediated degradation of cross-linked fibrin can be considered a biomarker of activation of coagulation and fibrinolysis^8^. D-Dimer has been found increased in COVID-19 patients^9^, and Zhou et al. demonstrated that the d-dimer levels on admission greater than 1 μg/mL were associated with an increase of in-hospital death^10^. Moreover, Xiang et al. demonstrated in a metanalysis including 16 observational studies higher mortality rate in patients with COVID-19-related coagulopathy (RR 10.86, 2.86 to 41.24, P < 0.001)^11^. Thus, the data related to coagulation parameters in different stages of COVID-19 disease may be of paramount importance to consider therapeutic prophylaxis or anticoagulation.

This study aims to summarize all available data on coagulation parameters in COVID-19 patients, particularly platelet count, Prothrombin Time (PT), D-dimer, and fibrinogen as suggested from the ISTH Interim Guidance on recognition and management of coagulopathy in COVID‐19^12^, and to perform a meta-analysis to assess differences in coagulopathy indices in different stages of COVID-19 disease.

## Methods

### Search strategy and study selection

An electronic search was performed within PubMed, Google Scholar and Scopus electronic databases between December 2019 (first confirmed Covid-19 case) up to April 6th, 2020. The following keywords were used for the search: “laboratory” or “coagulation” and “COVID-19” or “Coronavirus” or “SARS-CoV-2”. The English language was a limiting criterium for our analysis. All reports, including the search terms, were independently screened by two investigators for relevance and eligibility (I.L. and A.P.). Additionally, references from relevant articles were also manually scanned for additional studies. Where data were not available in the published study reports, authors were contacted, whenever possible, to supply missing information by email. The authors discussed their evaluation, and any disagreement was resolved through discussion and re-reading.

### Inclusion and exclusion criteria

Studies were considered eligible if the following statements were applying (a) they involved a study population with COVID-19 confirmed infection; (b) studies that stratify the risk of severe or fatal COVID-19; (c) they reported information on the difference of D-dimer values between two groups. Exclusion criteria were (just one was sufficient for study exclusion): non-original articles or articles with the number of patients less than 10, a duplicate publication with the same endpoint, endpoint measure not specified.

### Endpoints

The primary endpoint was the difference of D-dimer values between Non-Severe vs Severe disease and Survivors vs Non-Survivors. Moreover, results on additional coagulation parameters (platelets count, prothrombin time, activated partial thromboplastin time) were also analyzed.

### Data abstraction and management

Baseline characteristics and laboratory data were abstracted from the single studies through carefully scanning of the full article by two independent reviewers (I.L. and AP). Divergences were resolved by consensus. Moreover, the following data was extracted: year of publication, location, number of study patients, source type, peer-review process, study design, study groups. Selection and data abstraction were performed according to the MOOSE (Meta-analyses Of Observational Studies in Epidemiology) and PRISMA Checklist (Supplemental Tables S1, S2). The quality analysis of the selected studies was performed using the Agency for Healthcare Research and Quality (AHRQ) for cross-sectional study form (Supplemental Table S3).

### Statistical analysis

Mean and standard deviation were calculated from median and interquartile range (IQR), according to the formula reported by Wan et al.^13^ The summary measure used was the Standardized Mean Difference (SMD) with 95% confidence. Random-effects meta-analysis was used because high variability between studies was expected. Heterogeneity was evaluated using the I^2^ statistic. Cut-off values of 25%, 50%, and 75% indicated low, moderate, and high heterogeneity, respectively. Next, to explore potential sources of heterogeneity, we conducted a subgroup analysis between peer-reviewed/non-peer-reviewed articles. Finally, sensitivity analyses were performed by systematically removing each study, in turn, to explore its effect on outcome as previously described^14,15^. Publication bias was evaluated by the Egger test. Forest plots were used to graphically display the results of the meta-analysis, as already previously described^16,17^. All Analyses were performed using R Statistical Software (version 3.6.3; R Foundation for Statistical Computing, Vienna, Austria).

## Results

### Search results

**Figure 2:**
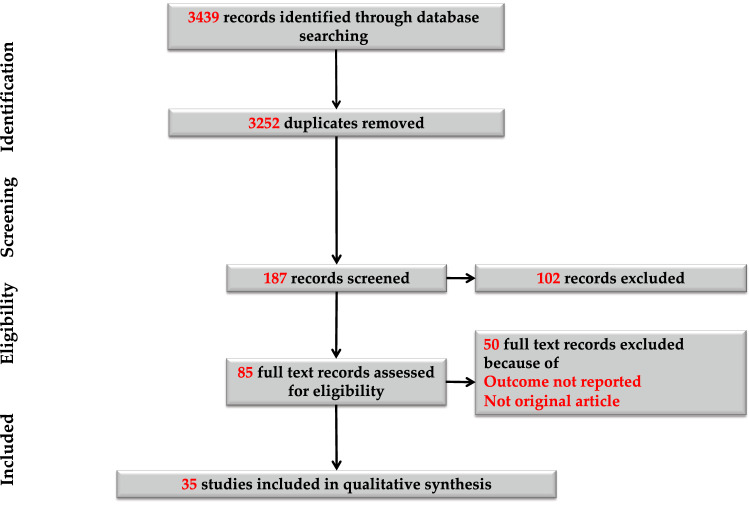
Flowchart depicting literature review and study selection.

Our search retrieved a total of 3439 entries, which were reduced to 3252 studies after duplicates removed. After the screening of 322 records, 290 studies were then excluded because they were not related to our research question. In the assessment of eligibility, further 20 studies were excluded because of: duplicate publication; outcome not reported; not original articles. Finally, a total of 35 studies were available for the analysis, including 6427 patients^9,10,18–50^. The study selection procedure is reported in detail in Fig. 2.

### Data on included studies

Since randomized trials were not currently available, only retrospective studies were included in the present meta-analysis. Table 1 summarizes the most relevant characteristics of the selected studies. Sixteen studies were peer-reviewed^9,10,18–31^, 19 were non-peer-reviewed^32–50^. Not surprisingly, quality assessment revealed a non-high study quality (Supplemental Table S1). Across the studies, patients were predominantly male and approximately one-fourth of patients had a history of cardiovascular disease. More details on patients’ characteristics are provided in Table 2.

**Table 1:** Characteristics of the studies included in the meta-analysis.

Study	Year	Location	N	Source type	Peer-reviewed	Study design	Study groups
Cai et al.^31^	2020	China	298	Journal Article	No	Retrospective study	Non-Severe vs Severe
Chen et al.^17^	2020	China	21	Journal Article	Yes	Retrospective study	Moderate vs Severe
Chen et al.^18^	2020	China	799	Journal Article	Yes	Retrospective study	Deaths vs Recovered Patients
Deng et al.^20^	2020	China	112	Journal Article	Yes	Retrospective study	Non-Severe vs Severe
Gao et al.^21^	2020	China	43	Journal Article	Yes	Retrospective study	Mild vs Severe
Han et al.^22^	2020	China	94	Journal Article	Yes	Retrospective study	Ordinary vs Severe/Critical
Huang et al.^9^	2020	China	41	Journal Article	Yes	Retrospective study	ICU care vs Non-ICU care
Huang et al.^33^	2020	China	125	Journal Article	No	Retrospective study	Mild vs Severe
Li et al.^34^	2020	China	134	Journal Article	No	Retrospective study	Non-Died Vs Died Moderate vs Severe/Critical
Li et al.^35^	2020	China	102	Journal Article	No	Retrospective study	Non-survivor vs Survivor
Li et al.^36^	2020	China	193	Journal Article	No	Retrospective study	Non-Severe vs Severe
Jiacheng et al.^37^	2020	China	122	Journal Article	No	Retrospective study	Common vs Severe
Jing et al.^38^	2020	China	40	Journal Article	No	Retrospective study	Mild vs Severe
Lu et al.^39^	2020	China	265	Journal Article	No	Retrospective study	Mild/Moderate vs Severe Critically Ill
Lu et al.^40^	2020	China	124	Journal Article	No	Retrospective study	Discharged vs Death
Luo et al.^41^	2020	China	403	Journal Article	No	Retrospective study	Recovered vs Died, Ordinary vs Severe/Critical
Ma et al.^42^	2020	China	84	Journal Article	No	Retrospective study	Non-Severe vs Severe
Qian et al.^43^	2020	China	91	Journal Article	No	Retrospective study	Mild vs Severe
Tang et al.^23^	2020	China	449	Journal Article	Yes	Retrospective study	Non-survivor vs Survivor
Wan et al.^24^	2020	China	135	Journal Article	Yes	Retrospective study	Mild vs Severe
Wang et al.^25^	2020	China	138	Journal Article	Yes	Retrospective study	ICU vs Non-ICU
Wang et al.^44^	2020	China	305	Journal Article	No	Retrospective study	Survivors vs Non-Survivors
Wang et al.^26^	2020	China	339	Journal Article	Yes	Retrospective study	Survival vs Dead
Wu et al.^28^	2020	China	201	Journal Article	Yes	Retrospective study	Patients with ARDS vs Patients without ARDS, Patients Alive vs Died Patients
Wu et al.^27^	2020	China	280	Journal Article	Yes	Retrospective study	Mild and Moderate type Patients vs Severe and Critically ill type Patients
Xu et al.^45^	2020	China	69	Journal Article	No	Retrospective study	Mild cases vs Severe or Critical cases
Zeng et al.^46^	2020	China	419	Journal Article	No	Retrospective study	ICU vs Non-ICU
Zhang et al.^47^	2020	China	48	Journal Article	No	Retrospective study	Survivors vs Non-Survivors
Zhang et al.^48^	2020	China	221	Journal Article	No	Retrospective study	Non-Severe vs Severe
Zhang et al.^29^	2020	China	140	Journal Article	Yes	Retrospective study	Non-Severe vs Severe
Zheng et al.^30^	2020	China	55	Journal Article	Yes	Retrospective study	Non-Severe vs Severe
Zheng et al.^49^	2020	China	52	Journal Article	No	Retrospective study	Severe vs Common
Zhou et al.^11^	2020	China	191	Journal Article	Yes	Retrospective study	Survivors vs Non-Survivors
Ying et al.^50^	2020	China	277	Journal Article	No	Retrospective study	Non-Severe vs Severe
Yulong et al.^31^	2020	China	17	Journal Article	Yes	Retrospective study	Non-Aggravation vs Aggravation Group

**Table 2:** Clinical characteristics of the patients included in the meta-analysis.

Study	Age Mean ± SD	Male N (%)	Hypertension N (%)	Smokers N (%)	Diabetes N (%)	CVD N (%)	COPD N (%)
Cai et al.^31^	47 ± 4.6	149 (50.0)	38 (12.8)	NA	19 (6.4)	11 (3.7)	NA
Chen et al.^17^	56 ± 3.7	17 (81.0)	5 (23.8)	NA	3 (14.3)	NA	NA
Chen et al.^18^	62 ± 4.3	171 (62.0)	97 (34.0)	12 (4.0)	47 (17.0)	23 (8.0)	18 (7.0)
Deng et al.^20^	65 ± 3.6	57 (50.9)	36 (32.1)	NA	19 (17.0)	15 (13.4)	4 (3.6)
Gao et al.^21^	43 ± 11.7	26 (60.0)	13 (30.2)	NA	7 (16.3)	3 (69.7)	8 (18.6)
Han et al.^22^	NA	NA	NA	NA	NA	NA	NA
Huang et al.^9^	49 ± 4.2	30 (73.0)	6 (15.0)	3 (7.0)	8 (20.0)	6 (8.0)	1 (2.0)
Huang et al.^33^	44 ± 18.5	63 (50.0)	20 (16.0)	NA	8 (6.4)	NA	NA
Li et al.^34^	61 ± 3.8	75 (56.0)	44 (32.8)	22 (16.4)	34 (25.3)	59 (44.0)	11 (8.2)
Li et al.^35^	5 7 ± 4.1	59 (58.0)	31 (30.0)	7 (7.0)	15 (15.0)	4 (4.0)	2 (2.0)
Li et al.^36^	67 ± 3.5	95 (49.0)	NA	NA	NA	70 (36.0)	NA
Liu Jiacheng et al.^37^	62 ± 3.8	72 (59.0)	50 (41.0)	5 (4.1)	15 (12.3)	2 (1.6)	2 (1.6)
Jing et al.^38^	48 ± 13.9	15 (37.5)	6 (15.0)	NA	6 (15.0)	NA	NA
Lu et al.^39^	NA	NA	52 (19.6)	NA	21 (7.9)	14 (5.3)	4 (1.5)
Lu et al.^40^	57 ± 12.6	61 (49.0)	41 (33.0)	17 (10.9)	14 (11.2)	15 (12.0)	6 (4.8)
Luo et al.^41^	56 ± 4.8	193 (47.9)	113 (28.0)	29 (7.2)	57 (14.1)	36 (8.9)	28 (6.9)
Ma et al.^42^	48 ± 3.3	48 (57.1)	12 (14.3)	7 (8.3)	10 (11.9)	5 (6.0)	5 (6.0)
Qian et al.^43^	50 ± 3.4	37 (40.7)	15 (16.4)	NA	8 (8.8)	3 (3.3)	NA
Tang et al.^23^	65 ± 12.0	268 (59.7)	177 (39.4)	NA	93 (20.7)	41 (9.1)	NA
Wan et al.^24^	47 ± 3.1	72 (53.3)	13 (9.6)	9 (6.7)	12 (8.9)	7 (5.2)	0 (0)
Wang et al.^25^	56 ± 4.3	75 (54.3)	43 (31.2)	NA	14 (10.1)	20 (14.5)	4 (2.9)
Wang et al.^44^	47 ± 15.1	142 (53.4)	45 (14.8)	NA	31 (10.2)	NA	NA
Wang et al.^26^	69 ± 1.8	166 (49.0)	138 (40.8)	NA	54 (16.0)	21 (15.7)	21 (6.2)
Wu et al.^28^	51 ± 2.8	128 (63.7)	39 (19.4)	NA	22 (10.9)	8 (4.0)	5 (2.5)
Wu et al.^27^	43 ± 19.0	151 (53.9)	NA	NA	NA	NA	1 (0.36)
Xu et al.^45^	57 ± 6.5	35 (50.7)	NA	5 (7.2)	NA	NA	NA
Zeng et al.^46^	46 ± 3.8	198 (47.2)	60 (14.3)	NA	24 (5.7)	18 (4.2)	5 (1.2)
Zhang al.^47^	70 ± 13.3	60 (68.9)	32 (51.8)	NA	10 (17.3)	13 (14.5)	NA
Zhang et al.^48^	55 ± 4.5	108 (48.9)	54 (24.4)	NA	22 (10.0)	22 (10.0)	6 (2.7)
Zhang et al.^29^	55 ± 10.0	71 (50.7)	42 (30.0)	NA	17 (12.1)	7 (5.0)	2 (1.4)
Zheng et al.^30^	59 ± 9.5	24 (43.6)	NA	NA	NA	NA	NA
Zheng et al.^49^	51 ± 15.9	23 (44.2)	12 (23.1)	NA	6 (11.5)	3 (5.8)	2 (3.8)
Zhou et al.^11^	56 ± 3.5	119 (62.0)	58 (30.0)	11 (6.0)	11 (19.0)	15 (8.0)	6 (3.0)
Ying et al.^50^	53 ± 15.3	170 (45.0)	133 (35.2)	NA	84 (22.2)	23 (6.1)	6 (1.6)
Yulong et al.^31^	42 ± 14 .0	6 (35.0)	NA	NA	NA	NA	NA

### Meta-analysis results

**Figure 3:**
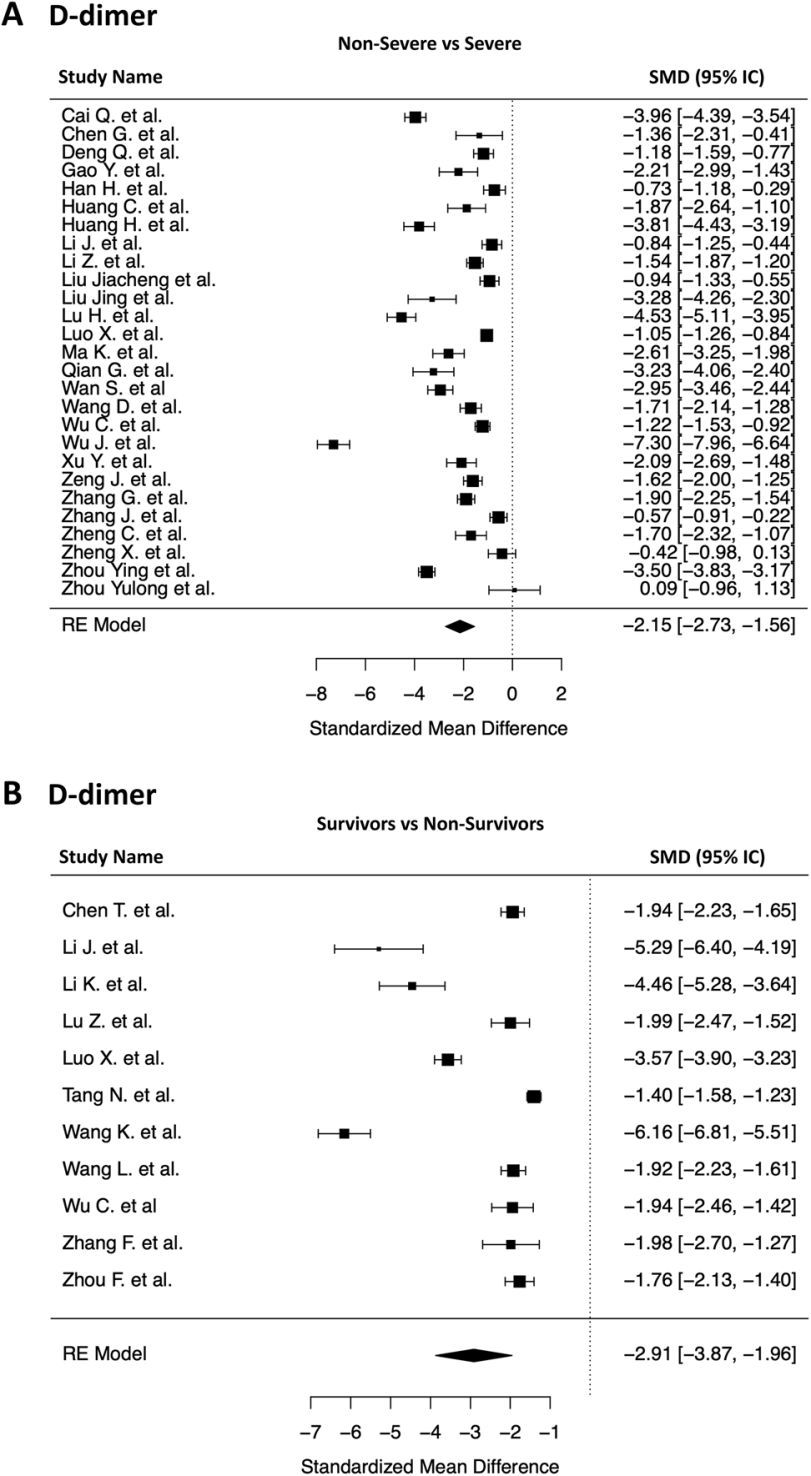
Forest plots of the standardized mean difference in d-dimer levels. (**A**) *Non severe vs Severe patients.* The black squares represent the pooled standardized mean difference effect size for each analysis while the left and right extremes of the squares represent the corresponding 95% confidence intervals for the pooled standardized mean difference effect size for each analysis. All analyses are based on a random-effects model. (**B**) *Survivors vs Non-Survivors.* The black squares represent the pooled standardized mean difference effect size for each analysis while the left and right extremes of the squares represent the corresponding 95% confidence intervals for the pooled standardized mean difference effect size for each analysis. All analyses are based on a random-effects model.

The primary analysis showed that mean d-dimer is significantly lower in COVID-19 patients with non-severe disease than in those with severe infection (SMD − 2.15 [− 2.73 to − 1.56], I^2^ 98%, P < 0.0001) (Fig. 3, panel A). Similarly, we found a much lower mean d-dimer in Survivors compared to Non-Survivors (SMD − 2.91 [− 3.87 to − 1.96], I^2^ 98%, P < 0.0001) (Fig. 3, panel B).

**Figure 4:**
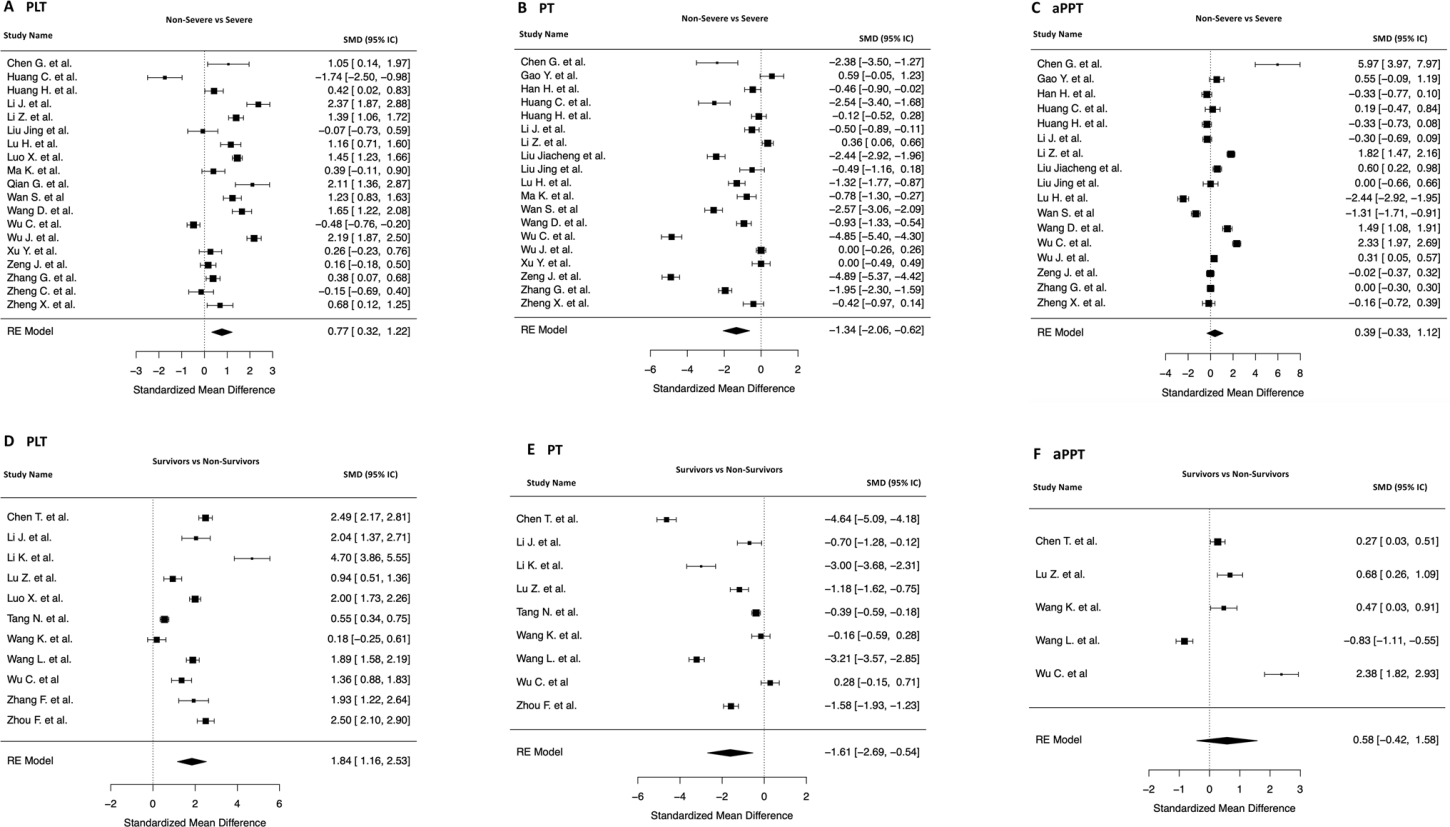
Forest plots of the standardized mean difference in platelets count (PLT), prothrombin time (PT) and activated partial thromboplastin time (aPTT). (**A**–**C**) Forest plots of the standard mean difference in PLT count, PT and aPTT between Non Severe and Severe patients. (**D**–**F**) Forest plots of the standard mean difference in PLT count, PT and aPTT between Survivors and Non-Survivors.

Additional analysis of platelet count showed higher mean PLT in Non-Severe patients than those observed in the Severe group (SMD 0.77 [0.32 to 1.22], I^2^ 96%, P < 0.001) (Fig. 4, panel A). Of note, a similar result was observed even when Survivors were compared to Non-Survivors (SMD 1.84 [1.16 to 2.53], I^2^ 97%, P < 0.0001) (Fig. 4, panel D).

Interestingly, shorter mean PT was found in both Non-Severe (SMD − 1.34 [− 2.06 to − 0.62], I^2^ 98%, P < 0.0002) (Fig. 4, panel B) and Survivors groups (SMD − 1.61 [− 2.69 to − 0.54], I^2^ 98%, P < 0.003) (Fig. 4, panel E) compared to Severe and Non-Survivor patients.

Whether no statistically significant differences were found in mean aPPT in both Non-Severe/Severe (SMD 0.39 [− 0.33 to 1.12], I^2^ 98%, P = 0.28) and Survivors/Non-Survivors (SMD 0.58 [− 0.42 to 1.58], I^2^ 97%, P = 0.26) (Fig. 4, panels C–F). Mean Fibrinogen was lower in both Non-Severe (SMD − 1.27 [− 1.86 to − 0.68], I^2^ 92%, P < 0.0001) (Supplementary Fig. S3, panel A) and Survivor patients (SMD − 1.16 [− 2.29 to − 0.04], I^2^ 94%, P = 0.04) (Supplementary Fig. S3, panel B). Even if few data was available in the studies included in our analysis regarding Fibrin Degradation Products (FDP) (Supplementary Fig. S4) and International Normalized Ratio (INR) (Supplementary Fig. S5) we found higher values for both parameters in Severe patients (SMD − 0.74 [− 1.46 to − 0.02], I^2^ 89%, P = 0.04 and SMD − 2.38 [− 5.13 to 0.36], I^2^ 98%, P = 0.08 respectively).

#### Subgroup and sensitivity analyses for the primary endpoint

As both peer-reviewed and non-peer-reviewed studies were included in this analysis (Table 1), we performed a subgroup analysis, revealing a similar result for both study types for the primary endpoint (peer-reviewed SMD − 1.90 [− 2.95 to − 0.84], I^2^ 98%, P < 0.001; non-peer-reviewed SMD − 2.34 [− 3.0 to − 1.68], I^2^ 97%, P < 0.0001) (Supplemental Fig. S1, panels A,B).

Moreover, sensitivity analysis performed by the leave-one-out approach showed that no single study had a substantial contribution to the pooled mean difference (Supplemental Fig. S2, panels A,B).

### Metaregression analysis

To evaluate the possible confounding effect of age on D-Dimer levels we performed a metaregression analysis using as covariate the ratio of mean age between the two groups (severe/non severe). No significant correlation between age and D-Dimer levels (SE 0.386; P = 0.772) were found at this additional analysis. Similar results have been obtained accepting as covariate the ratio of days from onset of symptoms to hospitalization between the two groups (SE 0.491; P = 0.274; Supplemental Fig. S5).

#### Publication bias

No evidence of publication bias was found by Egger’s test. The P values were: P = 0.07 for D-dimer, 0.81 for PLT, 0.13 for PT, and 0.10 for aPTT.

## Discussion

The major finding of the present meta-analysis is that higher levels of D-Dimer were found in patients with severe COVID-19. Finally, the mean platelet count is lower and mean prothrombin time more prolonged in Severe and Non-Survivor Covid-19 patients, supporting the concept that patients infected by COVID-19 may be at risk of developing disseminated intravascular coagulation (DIC). In fact, high d-dimer levels, low platelet count and prolonged PT are critical parameters of ISTH Criteria for DIC^3^. These findings corroborate the hypothesis that considers the COVID-19, in its most severe form, an endothelial disease^7^.

No differences in aPTT levels were found between the two groups, consistent with the results of a recently published metanalysis including 2277 patients^51^. In a study by Tang et al. from Wuhan, 71% of non-survivors from COVID-19 infection met the ISTH criteria for DIC compared to 0.4% of survivors. Elevated D-dimer values at admission and markedly increased over time were associated with a worse clinical outcome, likely reflecting coagulation activation from infection, cytokine storm and multiorgan failure^52,53^.

Lippi et al.^54^ showed in a brief letter reporting a pooled analysis of 4 studies that D-dimer is associated with the severity of COVID-19 disease. The mean difference of the four studies which reported continuous values (totaling 553 patients, 22% with severe disease) showed that D-dimer values are considerably higher in COVID-19 patients with severe disease than in those without (WMD: 2.97 mg/L; 95% CI 2.47–3.46 mg/L). Similarly, a recent metanalysis reported higher D-Dimer levels in patients with a more severe form of the disease (WMD 0.60, 0.49–0.71, I^2^ = 83.85%). Interestingly, this association seems to be independent from race and ethnicity^55^.

The obvious consideration is related to therapy with heparin to limit coagulopathy. However, to degrade pre‐existing fibrin in the lung it is essential to promote local fibrinolysis and a nebulizer form of tissue‐type plasminogen activator (tPA) to treat COVID‐19 has been recently proposed^56^.

Only one of the study included in our analysis investigated the effects of anticoagulation with low molecular weight heparin (LMWH) therapy on survival of Covid-19 patients, demonstrating that the use of anticoagulant therapy resulted in lower mortality in patients with severe coagulopathy with SIC score ≥ 4 (LMWH: 40.0% vs No-LMWH: 64.2%, P = 0.029) or D-dimer > sixfold of upper limit of normal (32.8% vs 52.4%, P = 0.017), but no overall benefit between heparin users and nonusers (30.3% vs 29.7%, P = 0.910)^23^. Moreover, a propensity-score matched retrospective study of 2785 COVID‐19 patients showed a significantly reduced cumulative incidence of in‐hospital death (HR 0.518 [0.308–0.872]) with the use of intermediate‐dose of anticoagulation compared to the only prophylactic‐dose and with the use of aspirin compared to no antiplatelet therapy (HR 0.522 [0.336–0.812])^57^^.^

Although coagulopathy recognizes multifactorial aetiology, our findings suggest that the worsening of coagulation parameters may indicate progressive severity of COVID-19 infection and may predict the need of more aggressive critical care and treatment. Thus, patients in the Intensive Care Unit (ICU) should have pharmacologic prophylaxis if there is not a contraindication and the benefit of heparin in COVID-19 patients in different stages of disease should be assessed. Clotting problems and antithrombotic therapy should be included in the daily COVID-19 management process, rather than just focusing on the infection. Furthermore, possible complications related to intravascular clotting should always be taken into account in the presence of worsening clinical conditions. Obviously, the risk of bleeding should always be considered in the individual patient when anticoagulant drugs are administered^58^.

Further studies are needed to define the role of coagulation indices in guiding the optimal timing to start antithrombotic drugs and the selection of patients in which this kind of therapies could have a greater prognostic impact.

### Limitations

Our study has some limitations. First, in the absence of randomized clinical trials, our analysis reported only data from retrospective and observational studies. Second, since there is significant heterogeneity, we used a random-effects model for all analyses. Third, the definition of the endpoints is variable in the different studies. Thus, we performed a subgroup analysis (Severe/Non Severe, Non Survivors/Survivors) to overcome this issue. Moreover, we took for our analysis laboratory data on admission in hospital of COVID-19 patients and this could represents a bias. However, the purpose of our study was to identify reliable biomarkers of severity on admission, in order to investigate the association of these biomarkers with disease severity.

### Conclusions

Results of the present meta-analysis demonstrate that Severe COVID-19 infection is associated with higher D-dimer values, lower platelet count and prolonged PT. This data suggests a possible role of disseminated intravascular coagulation in the pathogenesis of severe COVID-19 disease.

## Supplementary Information

Supplementary Information 1.

Supplementary Information 2.

## Data Availability

The datasets generated during and/or analysed during the current study are available from the corresponding author on reasonable request.
