# Supplementary material for: Differences in coagulopathy indices in patients with severe versus non-severe COVID-19: a meta-analysis of 35 studies and 6427 patients
Source: Sci Rep. 2021 May 17;11:10464. doi: 10.1038/s41598-021-89967-x (PMC8128869; doi:10.1038/s41598-021-89967-x)
Supplement: Supplementary file 1 — Supplementary Information 1. [file 41598_2021_89967_MOESM1_ESM.docx]

**Supplemental Figure 1. Subgroup Analysis (Peer-reviewed vs Non Peer-reviewed articles) for the primary endpoint.**

**Panel A.** Forest plot of the standard mean difference in D-dimer between Non-Severe and Severe patients.

**Panel B.** Forest plot of the standard mean difference in D-dimer between Survivors and Non-Survivors.

**Supplemental Figure 2. "Leave-one-out" Sensitivity Analysis**

**Panel A.** Sensitivity analysis performed by the leave-one-out approach showed that no single study had a substantial contribution to the pooled mean difference for the primary endpoint in Non-Severe vs Severe patients.

**Panel B.** Sensitivity analysis performed by the leave-one-out approach showed that no single study had a substantial contribution to the pooled mean difference for the primary endpoint in Survivors vs Non-Survivors.

**Supplemental Figure 3. Forest plots of the standardized mean difference in fibrinogen levels.**

**Panel A.** Forest plot of the standard mean difference in fibrinogen between Non-Severe and Severe patients.

**Panel B.** Forest plot of the standard mean difference in fibrinogen between Survivors and Non-Survivors.

**Supplemental Figure 4. Forest plots of the standardized mean difference in Fibrin degradation product (FDP) levels.**

Forest plot of the standard mean difference in FDS between Non-Severe and Severe patients.

**Supplemental Figure 5. Forest plots of the standardized mean difference in International Normalized Ratio (INR) levels.**

Forest plot of the standard mean difference in INR between Non-Severe and Severe patients.

**Supplemental Figure 6. Metaregression analysis.** Metaregression analysis performed to evaluate the effect of age and days from symptoms onset to hospitalization on D-Dimer levels. Both covariates had no significative impact on D-Dimer levels.
