# Supplementary material for: Differences in coagulopathy indices in patients with severe versus non-severe COVID-19: a meta-analysis of 35 studies and 6427 patients
Source: Sci Rep. 2021 May 17;11:10464. doi: 10.1038/s41598-021-89967-x (PMC8128869; doi:10.1038/s41598-021-89967-x)
Supplement: Supplementary file 2 — Supplementary Information 2. [file 41598_2021_89967_MOESM2_ESM.pdf]

Online supplement to:

Differences in coagulopathy indices in patients with severe versus non-severe COVID-19: A Meta-analysis of 35 Studies and 6427 Patients

Alberto Polimeni[1,2], MD, PhD, Isabella Leo, MD[1], Carmen Spaccarotella[1,2], MD, Annalisa Mongiardo[1], MD, Sabato Sorrentino[1,2], MD, PhD, Jolanda Sabatino[1,2], MD, PhD, Salvatore De Rosa[1,2], MD, PhD, Ciro Indolfi[1,2,3], MD

Affiliations:
Division of Cardiology[1] and Center for Cardiovascular Research[2], Department of Medical and Surgical Sciences, Magna Graecia University, Catanzaro, Italy. Mediterranea Cardiocentro, Naples, Italy [3].

Supplemental Table 1 - MOOSE Checklist

A reporting checklist for Authors, Editors, and Reviewers of Meta-analyses of Observational Studies. You must report the page number in your manuscript where you consider each of the items listed in this checklist. If you have not included this information, either revise your manuscript accordingly before submitting or note N/A.

Reporting Criteria	Reported (Yes/No)	Reported on Page No.
**Reporting of Background**		
Problem definition	Yes	4
Hypothesis statement	Yes	4
Description of Study Outcome(s)	Yes	7,8
Type of exposure or intervention used	Yes	5
Type of study design used	Yes	5
Study population	Yes	7
**Reporting of Search Strategy**		
Qualifications of searchers (eg, librarians and investigators)	Yes	5
Search strategy, including time period included in the synthesis and keywords	Yes	4,5
Effort to include all available studies, including contact with authors	Yes	5
Databases and registries searched	Yes	4
Search software used, name and version, including special features used (eg, explosion)	Yes	4
Use of hand searching (eg, reference lists of obtained articles)	Yes	5
List of citations located and those excluded, including justification	Yes	7
Method for addressing articles published in languages other than English	Yes	5
Method of handling abstracts and unpublished studies	Yes	5
Description of any contact with authors	Yes	5
**Reporting of Methods**		
Description of relevance or appropriateness of studies assembled for assessing the hypothesis to be tested	Yes	5
Rationale for the selection and coding of data (eg, sound clinical principles or convenience)	Yes	5
Documentation of how data were classified and coded (eg, multiple raters, blinding, and interrater reliability)	Yes	5
Assessment of confounding (eg, comparability of cases and controls in studies where appropriate	Yes	6

Reporting Criteria	Reported (Yes/No)	Reported on Page No.
Assessment of study quality, including blinding of quality assessors; stratification or regression on possible predictors of study results	Yes	5
Assessment of heterogeneity	Yes	6
Description of statistical methods (eg, complete description of fixed or random effects models, justification of whether the chosen models account for predictors of study results, dose-response models, or cumulative meta-analysis) in sufficient detail to be replicated	Yes	6
Provision of appropriate tables and graphics	Yes	5,7,8
**Reporting of Results**		
Table giving descriptive information for each study included	Yes	7
Results of sensitivity testing (eg, subgroup analysis)	Yes	8
Indication of statistical uncertainty of findings	Yes	7,8
**Reporting of Discussion**		
Quantitative assessment of bias (eg, publication bias)	Yes	6
Justification for exclusion (eg, exclusion of non–English-language citations)	Yes	5
Assessment of quality of included studies	Yes	5
**Reporting of Conclusions**		
Consideration of alternative explanations for observed results	Yes	10
Generalization of the conclusions (ie, appropriate for the data presented and within the domain of the literature review)	Yes	9,10
Guidelines for future research	Yes	10
Disclosure of funding source	Yes	1

Once you have completed this checklist, please save a copy and upload it as part of your submission. DO NOT include this checklist as part of the main manuscript document. It must be uploaded as a separate file.

PRISMA 2009 Checklist

Supplemental Table 2 – Prisma Checklist

Section/topic	#	Checklist item	Reported on page #
**TITLE**			
Title	1	Identify the report as a systematic review, meta-analysis, or both.	1
**ABSTRACT**			
Structured summary	2	Provide a structured summary including, as applicable: background; objectives; data sources; study eligibility criteria, participants, and interventions; study appraisal and synthesis methods; results; limitations; conclusions and implications of key findings; systematic review registration number.	2
**INTRODUCTION**			
Rationale	3	Describe the rationale for the review in the context of what is already known.	4
Objectives	4	Provide an explicit statement of questions being addressed with reference to participants, interventions, comparisons, outcomes, and study design (PICOS).	4
**METHODS**			
Protocol and registration	5	Indicate if a review protocol exists, if and where it can be accessed (e.g., Web address), and, if available, provide registration information including registration number.	NA
Eligibility criteria	6	Specify study characteristics (e.g., PICOS, length of follow-up) and report characteristics (e.g., years considered, language, publication status) used as criteria for eligibility, giving rationale.	5
Information sources	7	Describe all information sources (e.g., databases with dates of coverage, contact with study authors to identify additional studies) in the search and date last searched.	5
Search	8	Present full electronic search strategy for at least one database, including any limits used, such that it could be repeated.	5
Study selection	9	State the process for selecting studies (i.e., screening, eligibility, included in systematic review, and, if applicable, included in the meta-analysis).	5
Data collection process	10	Describe method of data extraction from reports (e.g., piloted forms, independently, in duplicate) and any processes for obtaining and confirming data from investigators.	5,6
Data items	11	List and define all variables for which data were sought (e.g., PICOS, funding sources) and any assumptions and simplifications made.	5,6
Risk of bias in individual studies	12	Describe methods used for assessing risk of bias of individual studies (including specification of whether this was done at the study or outcome level), and how this information is to be used in any data synthesis.	6
Summary measures	13	State the principal summary measures (e.g., risk ratio, difference in means).	6
Synthesis of results	14	Describe the methods of handling data and combining results of studies, if done, including measures of consistency (e.g., I^2) for each meta-analysis.	6

PRISMA 2009 Checklist

Section/topic	#	Checklist item	Reported on page #
Risk of bias across studies	15	Specify any assessment of risk of bias that may affect the cumulative evidence (e.g., publication bias, selective reporting within studies).	6
Additional analyses	16	Describe methods of additional analyses (e.g., sensitivity or subgroup analyses, meta-regression), if done, indicating which were pre-specified.	6
**RESULTS**			
Study selection	17	Give numbers of studies screened, assessed for eligibility, and included in the review, with reasons for exclusions at each stage, ideally with a flow diagram.	7
Study characteristics	18	For each study, present characteristics for which data were extracted (e.g., study size, PICOS, follow-up period) and provide the citations.	7
Risk of bias within studies	19	Present data on risk of bias of each study and, if available, any outcome level assessment (see item 12).	8
Results of individual studies	20	For all outcomes considered (benefits or harms), present, for each study: (a) simple summary data for each intervention group (b) effect estimates and confidence intervals, ideally with a forest plot.	7,8
Synthesis of results	21	Present results of each meta-analysis done, including confidence intervals and measures of consistency.	7,8
Risk of bias across studies	22	Present results of any assessment of risk of bias across studies (see Item 15).	8
Additional analysis	23	Give results of additional analyses, if done (e.g., sensitivity or subgroup analyses, meta-regression [see Item 16]).	8
**DISCUSSION**			
Summary of evidence	24	Summarize the main findings including the strength of evidence for each main outcome; consider their relevance to key groups (e.g., healthcare providers, users, and policy makers).	9,10
Limitations	25	Discuss limitations at study and outcome level (e.g., risk of bias), and at review-level (e.g., incomplete retrieval of identified research, reporting bias).	10
Conclusions	26	Provide a general interpretation of the results in the context of other evidence, and implications for future research.	10
**FUNDING**			
Funding	27	Describe sources of funding for the systematic review and other support (e.g., supply of data); role of funders for the systematic review.	14

From: Moher D, Liberati A, Tetzlaff J, Altman DG, The PRISMA Group (2009). Preferred Reporting Items for Systematic Reviews and Meta-Analyses: The PRISMA Statement. PLoS Med 6(7): e1000097. doi:10.1371/journal.pmed1000097

For more information, visit: www.prisma-statement.org.

Supplemental Table 3 - QUALITY ASSESSMENT AHRQ

	1	2	3	4	5	6	7	8	9	10	11
Cai Q.	Yes	Yes	Yes	Unclear	Unclear	Yes	No	Yes	No	No	No
Chen G.	Yes	Yes	Yes	Unclear	Unclear	Yes	No	Yes	No	No	No
Chen T.	Yes	Yes	Yes	Unclear	Unclear	Yes	No	No	No	Yes	No
Deng Q.	Yes	Yes	Yes	Unclear	Unclear	No	No	No	No	No	No
Gao Y.	Yes	Yes	Yes	Unclear	Unclear	No	No	Yes	No	No	No
Han H.	Yes	Yes	Yes	Unclear	Unclear	No	No	No	No	No	No
Huang C.	Yes	Yes	Yes	Unclear	Unclear	Yes	No	No	No	No	No
Huang H.	Yes	Yes	Yes	Unclear	Unclear	Yes	Yes	Yes	No	No	No
Li J.	Yes	Yes	Yes	Unclear	Unclear	No	Yes	Yes	No	No	No
Li K.	Yes	Yes	Yes	Unclear	Unclear	Yes	Unclear	Yes	No	No	No
Li Z.	Yes	Yes	Yes	Unclear	Unclear	Yes	No	No	No	No	No
Liu Jiacheng	Yes	Yes	Yes	Unclear	Unclear	No	No	Yes	No	No	No
Liu Jing	Yes	Yes	Yes	Unclear	Unclear	No	Yes	Yes	No	No	No
Lu H.	Yes	Yes	Yes	Unclear	Unclear	No	No	No	No	No	No
Lu Z.	Yes	Yes	Yes	Yes	Unclear	Yes	Yes	Yes	Yes	No	No
Luo X.	Yes	Yes	Yes	Unclear	Unclear	No	Yes	No	No	No	No
Ma K.	Yes	Yes	Yes	Yes	Unclear	Yes	Yes	Yes	No	Yes	No
Qian G.	Yes	Yes	Yes	Unclear	Unclear	No	No	No	Yes	No	No
Tang N.	Yes	Yes	Yes	Yes	Unclear	No	No	Yes	No	No	No
Wan S.	Yes	Yes	Yes	Unclear	Unclear	No	No	Yes	No	No	No
Wang D.	Yes	Yes	Yes	Yes	Unclear	No	No	No	No	No	No
Wang K.	Yes	Yes	Yes	Unclear	Unclear	No	Yes	Yes	No	Yes	No
Wang L.	Yes	Yes	Yes	Yes	Unclear	Yes	Yes	No	No	No	No
Wu C.	Yes	Yes	Yes	Unclear	Unclear	No	No	No	Yes	Yes	No
Wu J.	Yes	Yes	Yes	Unclear	Unclear	No	No	Yes	No	No	No
Xu Y.	Yes	Yes	Yes	Yes	Unclear	No	Yes	No	No	No	No
Zeng J.	Yes	Yes	Yes	Unclear	Unclear	No	No	No	No	No	No
Zhang F.	Yes	Yes	Yes	Yes	Unclear	No	Yes	No	No	No	No
Zhang G.	Yes	Yes	Yes	Unclear	Unclear	No	No	No	Yes	No	No
Zhang J.	Yes	Yes	Yes	Unclear	Unclear	Yes	No	Yes	No	No	No
Zheng C.	Yes	Yes	Yes	Unclear	Unclear	No	No	No	No	No	No
Zheng X.	Yes	Yes	Yes	Unclear	Unclear	Yes	Yes	Yes	No	Yes	No
Zhou F.	Yes	Yes	Yes	Unclear	Unclear	Yes	Yes	Yes	No	No	No
Zhou Ying	Yes	Yes	Yes	Unclear	Unclear	Yes	Yes	Yes	Yes	No	No
Zhou Yulong	Yes	Yes	Yes	Unclear	Unclear	No	No	Yes	No	No	No

1) Define the source of information; 2) List inclusion and exclusion criteria for exposed and unexposed subjects (cases and controls) or refer to previous publications;3) Indicate time period used for identifying patients;4) Indicate whether or not subjects were consecutive if not population-based;5) Indicate if evaluators of subjective components of study were masked to other aspects of the status of the participants;6) Describe any assessments undertaken for quality assurance purposes (e.g., test/retest of primary outcome measurements);7) Explain any patient exclusions from analysis;8) Describe how confounding was assessed and/or controlled.;9) If applicable, explain how missing data were handled in the analysis;10) Summarize patient response rates and completeness of data collection;11) Clarify what follow-up, if any, was expected and the percentage of patients for which incomplete data or follow-up was obtained

Supplemental Figure 1

A D-dimer

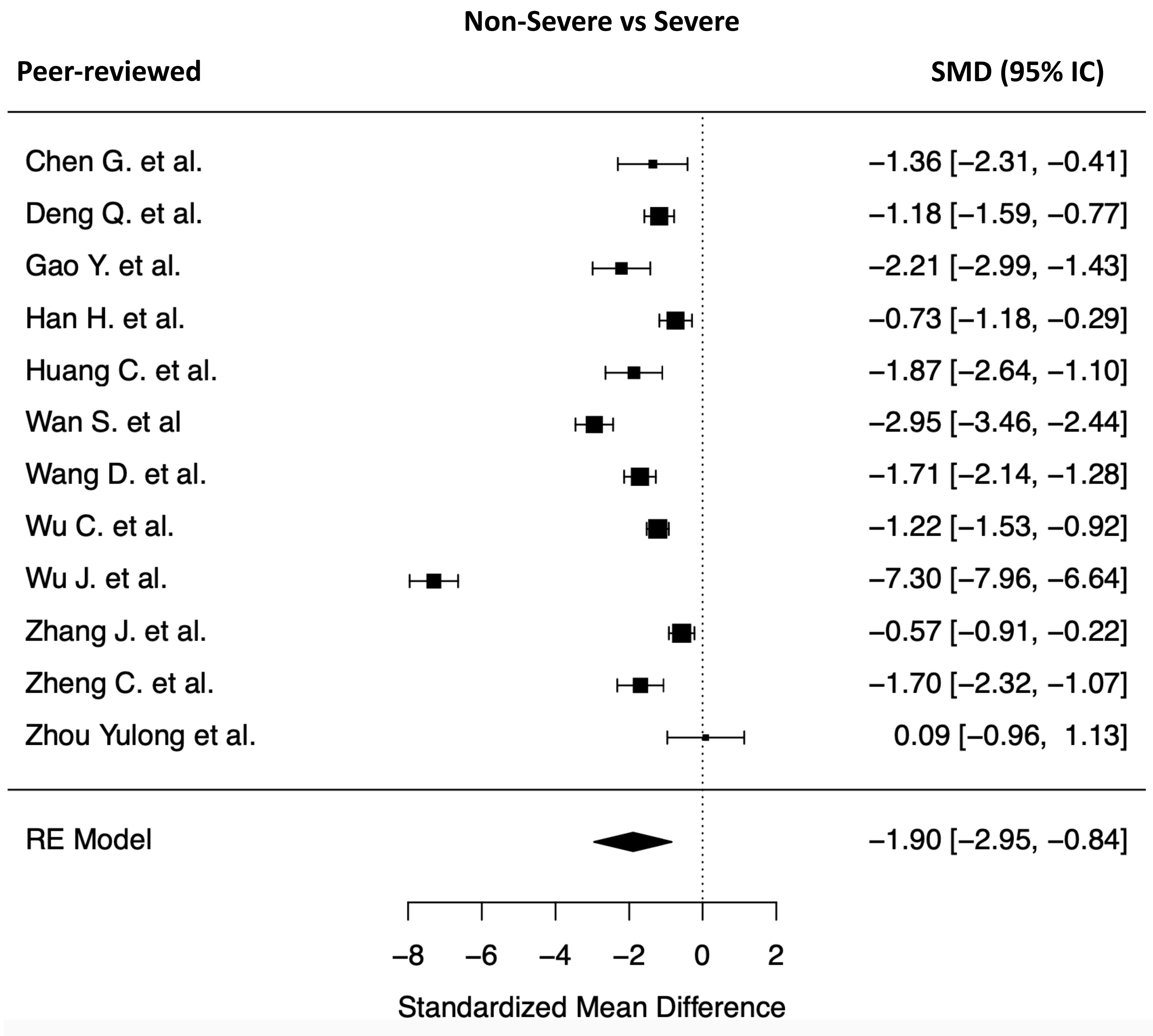

B D-dimer

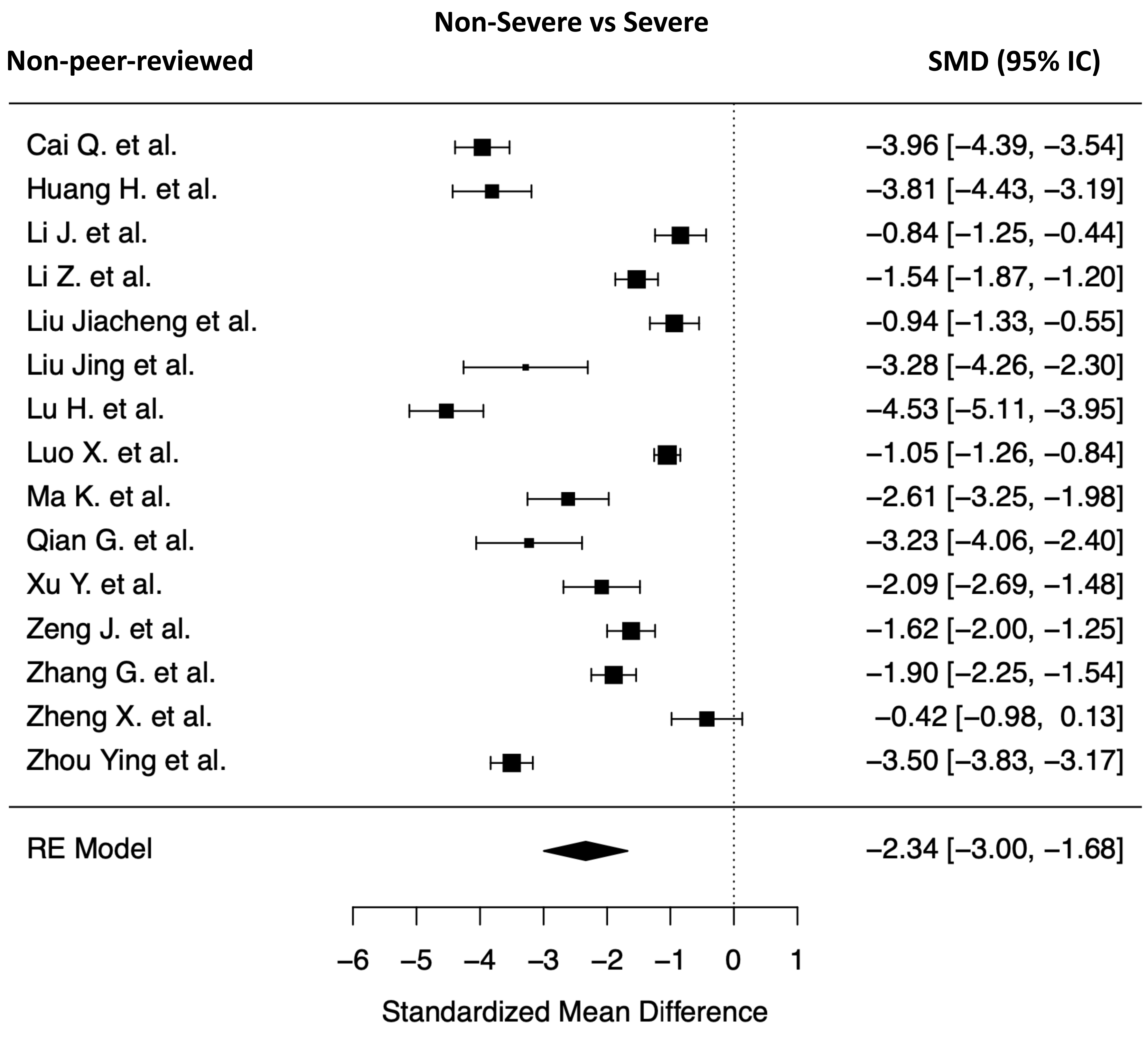

Supplemental Figure 2

A Sensitivity Analysis for D-dimer outcome

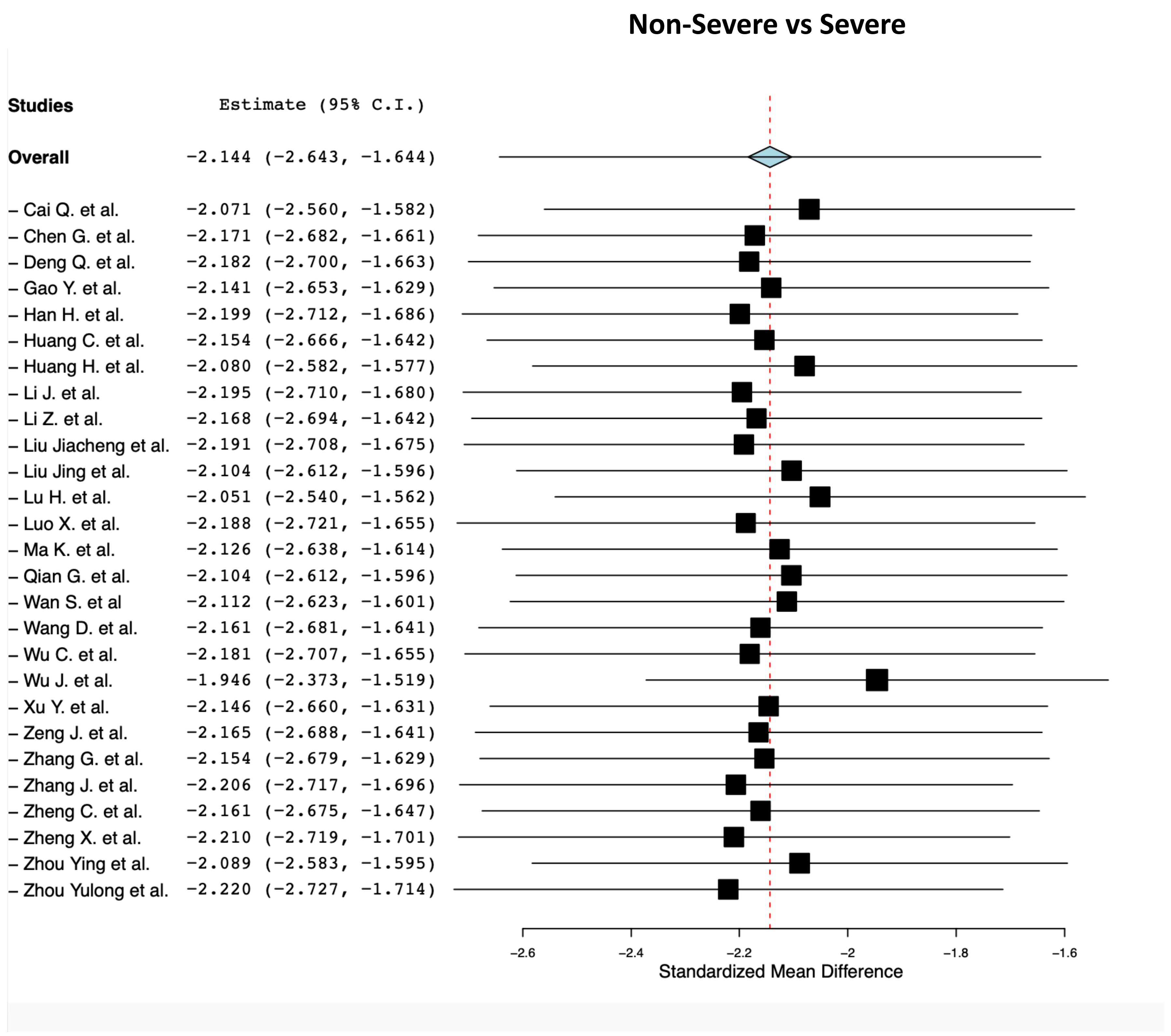

B Sensitivity Analysis for D-dimer outcome

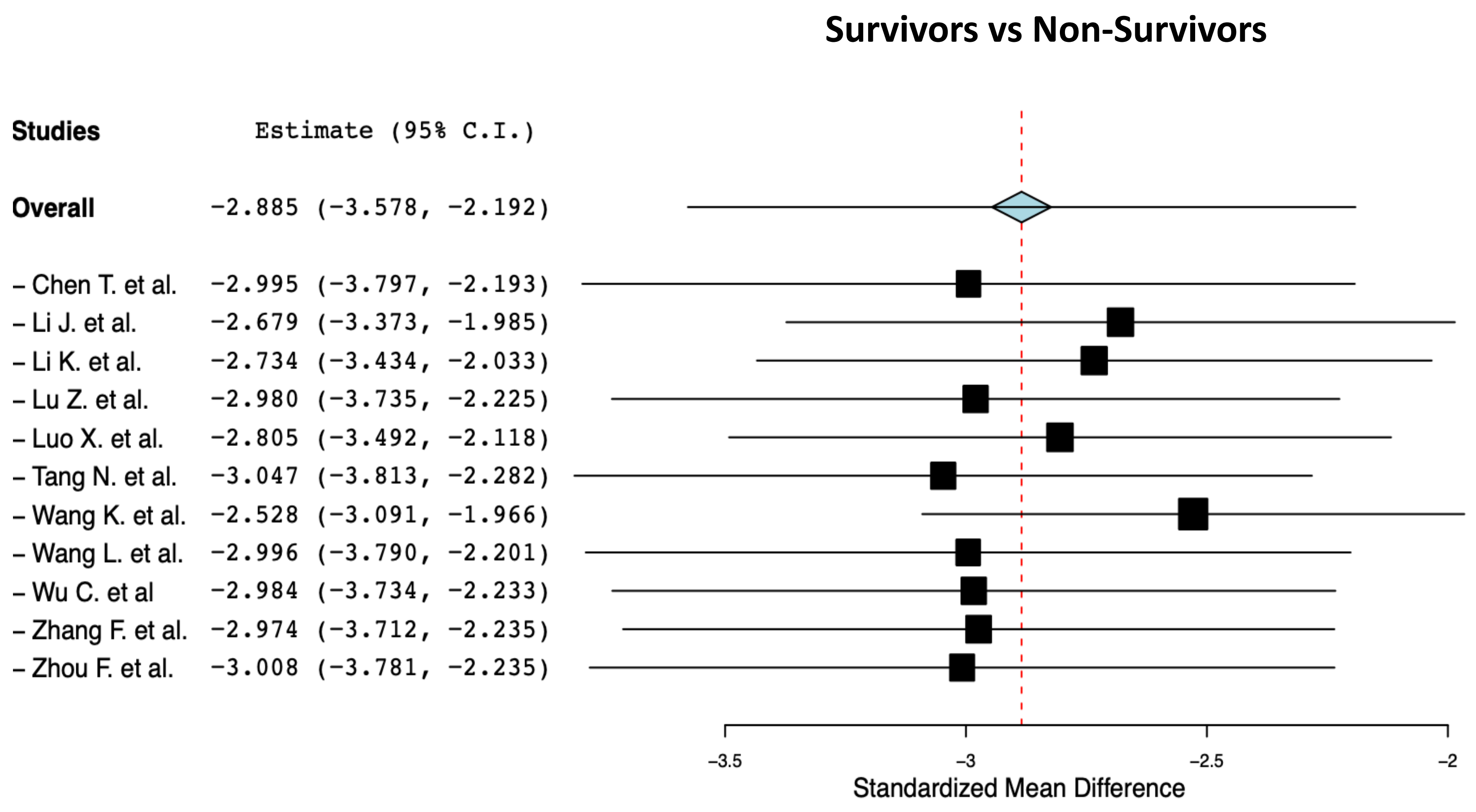

Supplemental Figure 3

A Fibrinogen

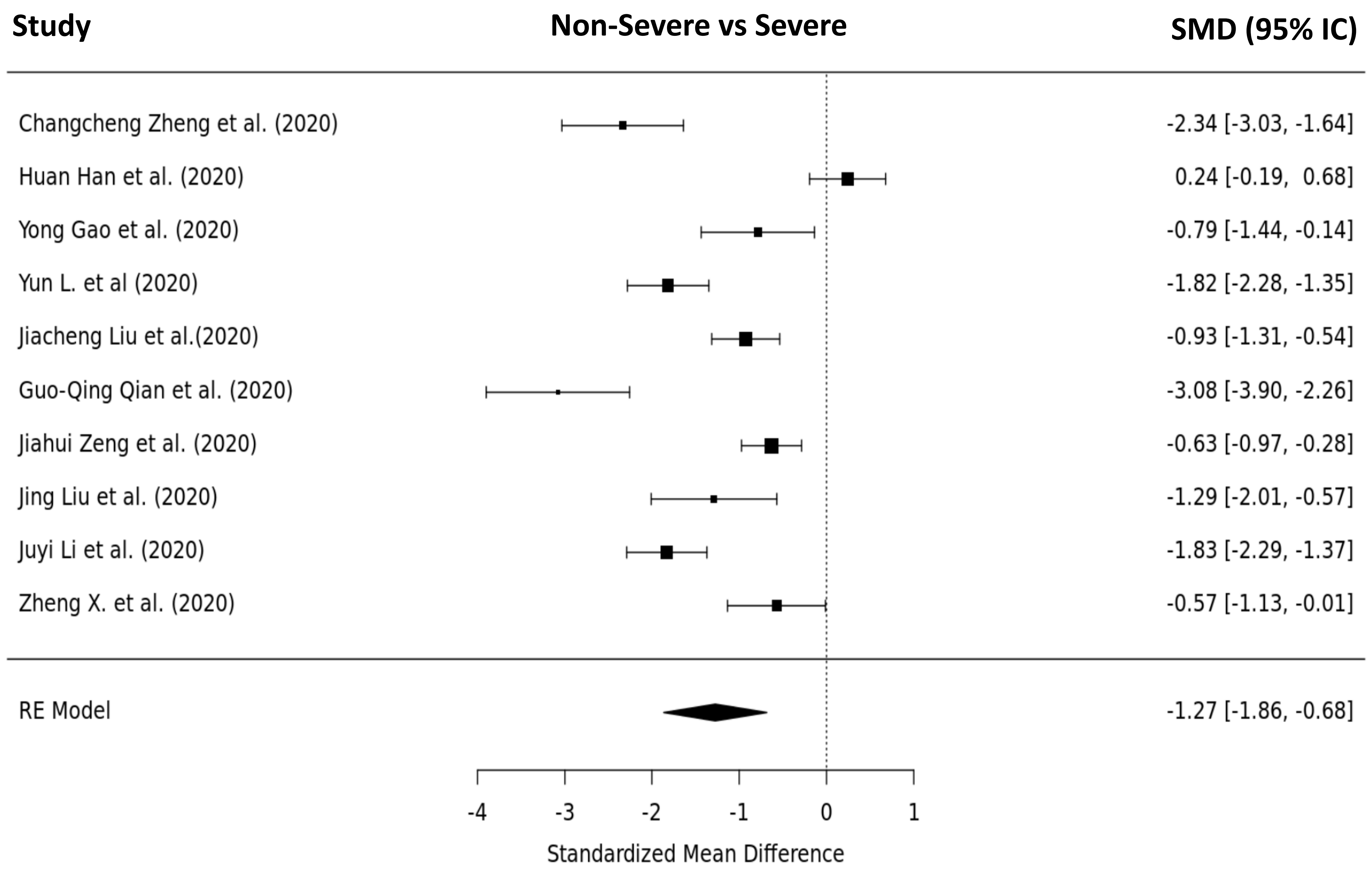

B Fibrinogen

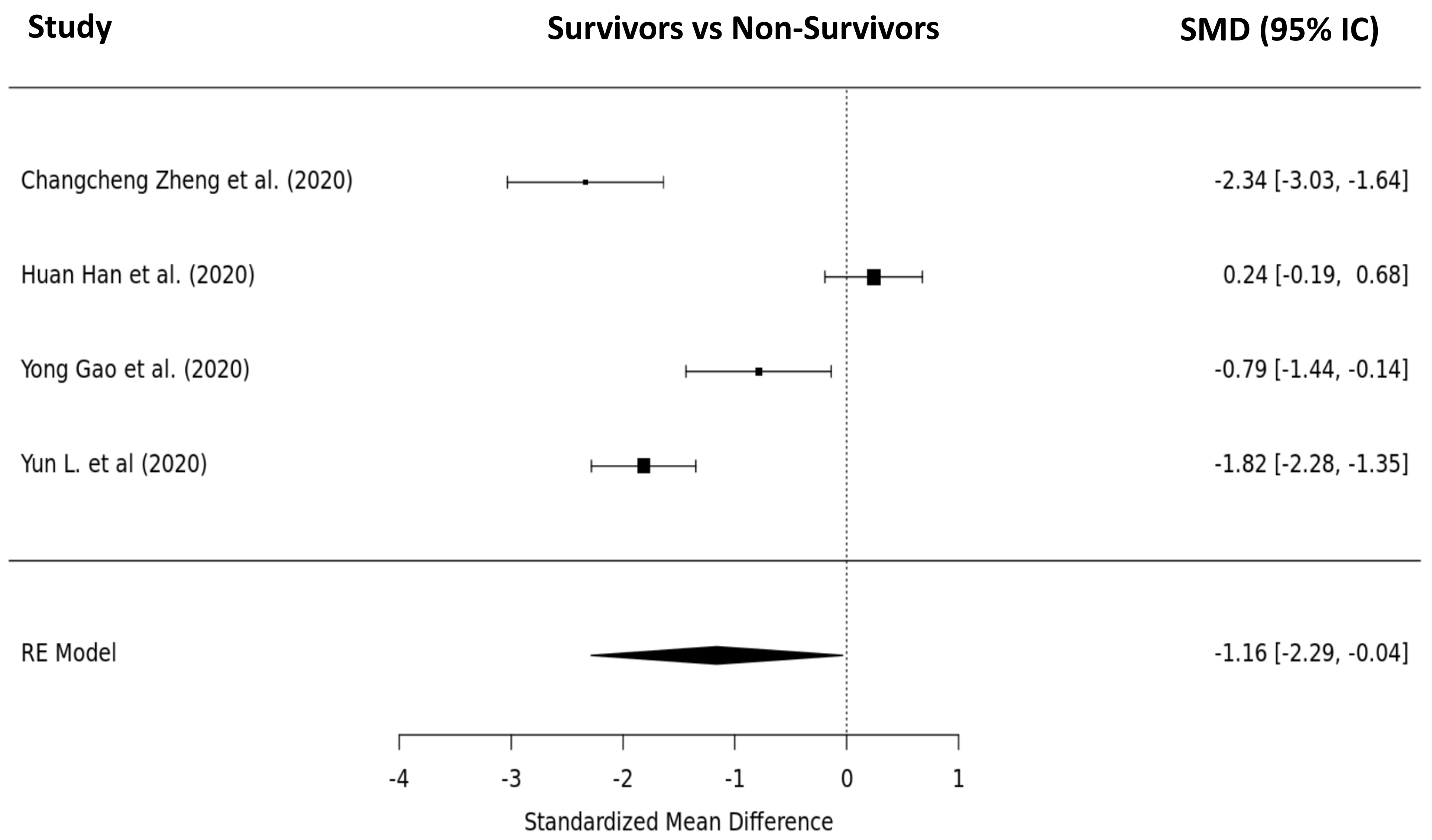

Supplemental Figure 4

Fibrin Degradation Product

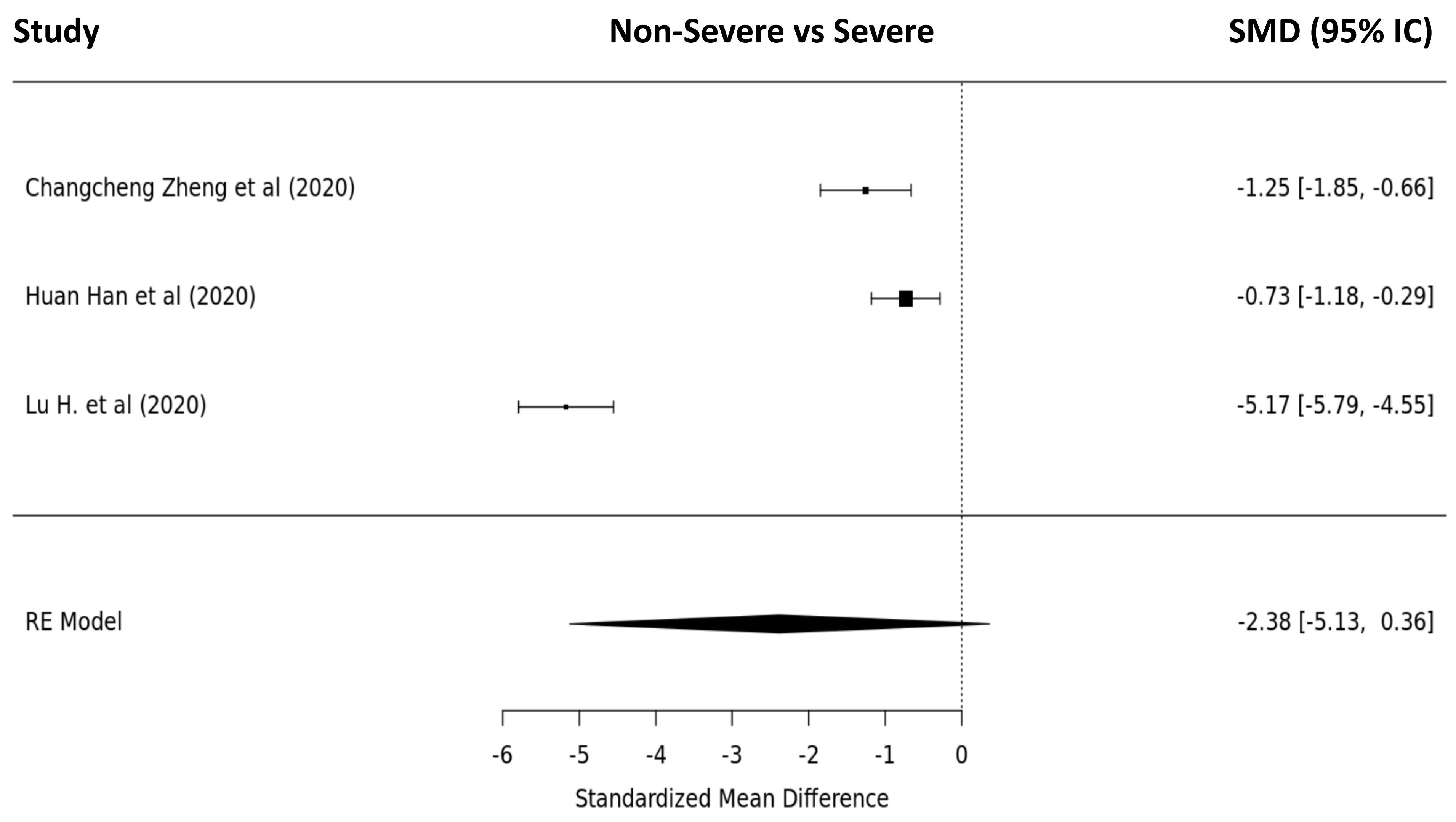

Supplemental Figure 5

International Normalized Ratio (INR)

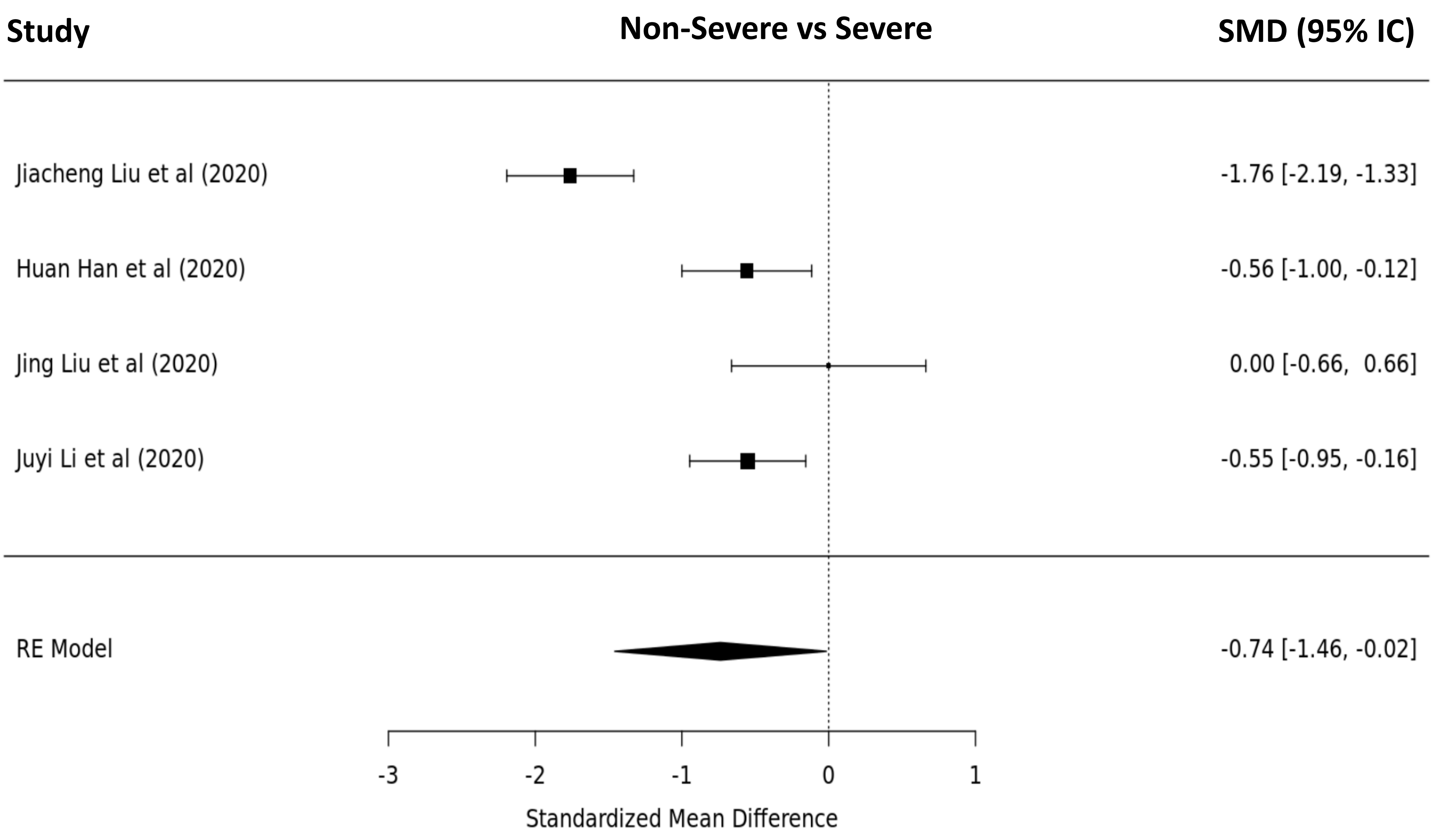

Supplemental Figure 6

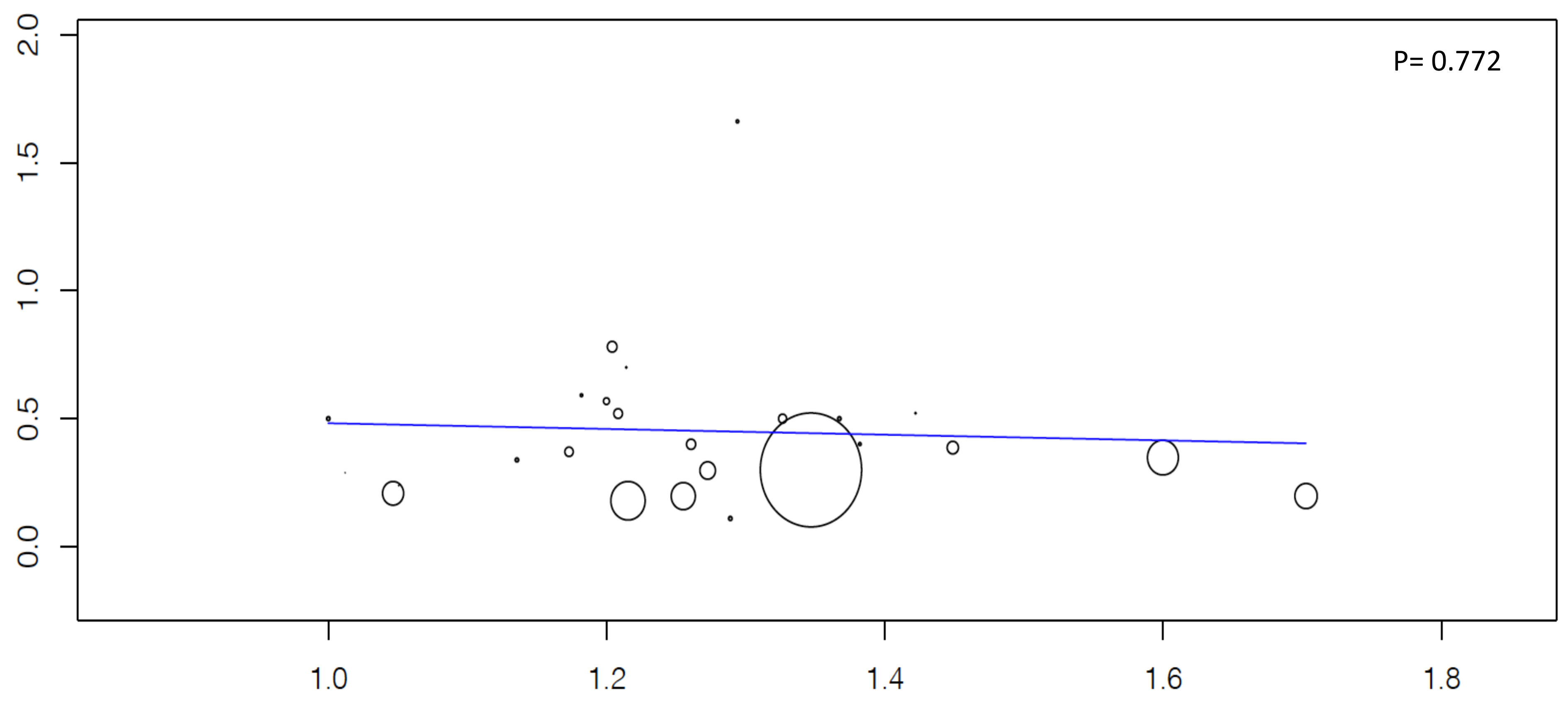

Mean age in
severe /non-severe group

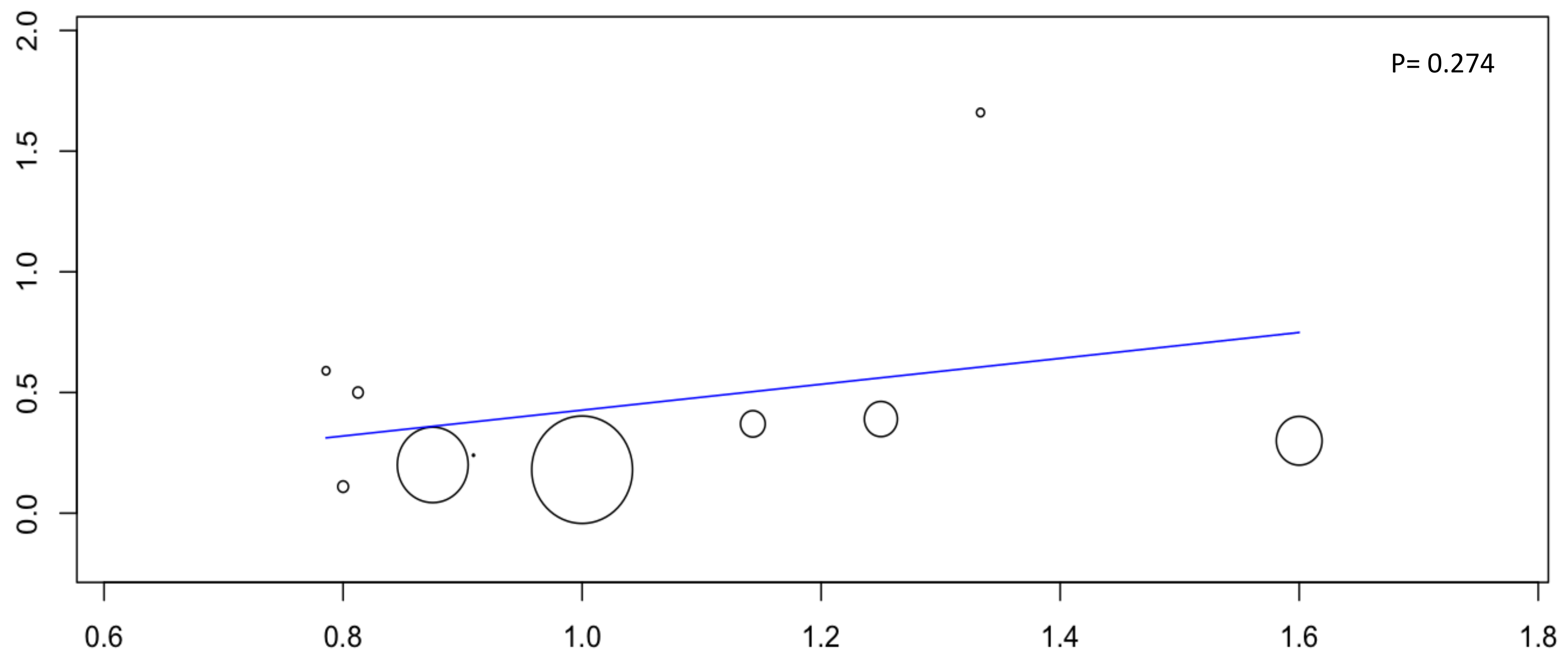

Mean days from onset of symptoms
to hospitalization
in severe/non-severe group
